# The lncRNA H19-Derived MicroRNA-675 Promotes Liver Necroptosis by Targeting FADD

**DOI:** 10.3390/cancers13030411

**Published:** 2021-01-22

**Authors:** Rona Harari-Steinfeld, Maytal Gefen, Alina Simerzin, Elina Zorde-Khvalevsky, Mila Rivkin, Ezra Ella, Tomer Friehmann, Mordechay Gerlic, Jessica Zucman-Rossi, Stefano Caruso, Mélissa Leveille, Jennifer L. Estall, Daniel S. Goldenberg, Hilla Giladi, Eithan Galun, Zohar Bromberg

**Affiliations:** 1 The Goldyne Savad Institute of Gene and Cell Therapy, Hadassah Hebrew University Medical Center, Ein Karem, P.O.B. 12000, Jerusalem 9112001, Israel; rona.harari@refugebiotech.com (R.H.-S.); maytal.gefen@mail.huji.ac.il (M.G.); Alina_simerzin@hms.harvard.edu (A.S.); Elina@hadassah.org.il (E.Z.-K.); lrivkin@hadassah.org.il (M.R.); Ezra.Ella@mail.huji.ac.il (E.E.); Tomer.friehmann@mail.huji.ac.il (T.F.); goldenberg@hadassah.org.il (D.S.G.); giladi@hadassah.org.il (H.G.); zoharb@hadassah.org.il (Z.B.); 2 Department of Clinical Microbiology and Immunology, Sackler Faculty of Medicine, Tel Aviv University, Tel Aviv 69978, Israel; mgerlic@post.tau.ac.il; 3 Centre de Recherche des Cordeliers, Sorbonne Université, Université de Paris, INSERM, Functional Genomics of Solid Tumors Laboratory, Equipe Labellisée Ligue Nationale Contre le Cancer, Labex OncoImmunology, F-75006 Paris, France; Jessica.zucman-rossi@inserm.fr (J.Z.-R.); stefano.caruso@inserm.fr (S.C.); 4 Assistance Publique Hopitaux de Paris, AP-HP, Hopital Européen Georges Pompidou, HEGP, Service d’Oncologie, F-75015 Paris, France; 5 Cardiovascular and Metabolic Disease Division, Institut de Recherches Cliniques de Montreal (IRCM), 110 Ave des Pins Ouest, Montreal, QC H2W 1R7, Canada; meli.leveille04@gmail.com (M.L.); jennifer.estall@ircm.qc.ca (J.L.E.)

**Keywords:** hepatocellular carcinoma, liver inflammation, necrosis, apoptosis

## Abstract

**Simple Summary:**

Liver cancer develops mostly in a chronically inflamed liver. The inflammation process can enhance or suppress the development of liver cancer. *H19* is a noncoding RNA that is upregulated in inflamed livers. The role of *H19* in liver cancer was intensely investigated, but the reported findings are conflicting. Some reported that *H19* is a tumor suppressor and others that it has oncogenic properties. To understand the contribution of *H19* to liver cancer development, we investigated miR-675, which is generated from the first exon of the *H19* RNA message. Interestingly, we found that miR-675 suppresses liver cancer cell growth by inducing cell death. Following our investigation of the mechanism of this killing effect, we established that miR-675 represses the protein called Fas-associated protein with death domain (FADD) and that this repression leads to the development of a specific type of necrosis named necroptosis. These findings can have future therapeutic implications.

**Abstract:**

The *H19*-derived microRNA-675 (miR-675) has been implicated as both tumor promoter and tumor suppressor and also plays a role in liver inflammation. We found that miR-675 promotes cell death in human hepatocellular carcinoma (HCC) cell lines. We show that Fas-associated protein with death domain (FADD), a mediator of apoptotic cell death signaling, is downregulated by miR-675 and a negative correlation exists between miR-675 and FADD expression in mouse models of HCC (*p* = 0.014) as well as in human samples (*p* = 0.017). We demonstrate in a mouse model of liver inflammation that overexpression of miR-675 promotes necroptosis, which can be inhibited by the necroptosis-specific inhibitor Nec-1/Nec-1s. miR-675 induces the level of both p-MLKL (Mixed Lineage Kinase Domain-Like Pseudokinase) and RIP3 (receptor-interacting protein 3), which are key signaling molecules in necroptosis, and enhances MLKL binding to RIP3. miR-675 also inhibits the levels of cleaved caspases 8 and 3, suggesting that miR-675 induces a shift from apoptosis to a necroptotic cellular pathway. In conclusion, downregulation of FADD by miR-675 promotes liver necroptosis in response to inflammatory signals. We propose that this regulation cascade can stimulate and enhance the inflammatory response in the liver, making miR-675 an important regulator in liver inflammation and potentially also in HCC.

## 1. Introduction

Cell death has been shown to be involved in liver diseases, including inflammatory, infectious, metabolic, and hepatocellular carcinoma (HCC) [1,2]. In recent years, another distinct form of cell death has been characterized, namely necroptosis, also referred to as programmed necrosis. This newly described mechanism of cell death occurs in a regulated manner in response to specific stresses, causing specific signaling, but shares many of the cellular attributes of necrosis. While both apoptosis and necroptosis lead to cell death, the effect of the two on the surrounding tissue microenvironment and subsequent immune response is markedly different [3,4]. Unlike apoptosis, necroptosis is highly inflammatory due to the rupture of cellular membrane and the “spilling” of cellular contents, including many danger-associated compounds into the tissue.

Necroptosis is mediated by the kinase receptor-interacting proteins RIP1 and RIP3 that form a complex called the necrosome [5,6]. RIP1 phosphorylates RIP3, which, in turn, phosphorylates MLKL (Mixed Lineage Kinase Domain-Like Pseudokinase), which is recruited to the necrosome [7]. Phosphorylation leads to the oligomerization of MLKL, which disrupts membrane integrity and subsequently results in cell death [8].

Fas-associated protein with death domain (FADD) is a critical adaptor protein for death receptor-mediated apoptosis. In addition to its role in apoptosis, FADD is implicated in different non-apoptotic processes, including a role in the regulation of necroptosis. Activated caspase-8, which depends on FADD, cleaves the RIP1 and RIP3 kinases and, thus, is responsible for inhibiting necroptosis [9]. Accordingly, cells deficient in FADD facilitate necroptosis as they are unable to recruit and activate procaspase-8 [10].

*H19* is an imprinted, maternally expressed gene that is transcribed into a long noncoding RNA (lncRNA). In the liver, *H19* is expressed only at pre-natal stages but is also expressed in primary liver tumors. However, its role in tumor initiation and progression has been a subject of controversy [11]. It was also shown that *H19* levels are increased in fibrotic and cirrhotic livers [12]. *H19* serves as a precursor of miR-675, which is embedded in its first exon [13]. miR-675 is mainly referred to as an oncomiR (cancer-promoting miRNA) and is upregulated in a subset of HCC cases, as well as many other cancers, and is considered a bad prognostic marker in HCC [14,15,16,17].

In this study, we validated that the cell death facilitator FADD gene is a target of miR-675. We demonstrate that the downregulation of FADD by miR-675 promotes necroptosis both in vitro and in vivo. We believe that miR-675 may play a complex role in the development of inflammation-induced HCC by manipulating cell death through a necroptotic pathway.

## 2. Results

### 2.1. Positive Correlation between H19 and miR-675 in Human and Mouse Hepatocellular Carcinoma (HCC)

**Figure 1:**
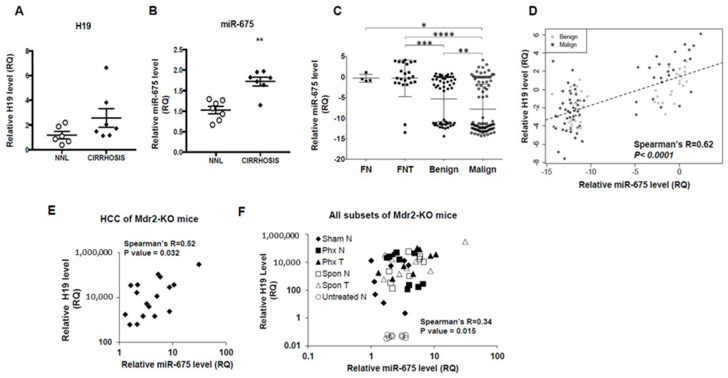
Positive H19/miR-675 correlation in human and mouse liver samples. (**A**,**B**) Quantitative Reverse Transcription-Polymerase Chain Reaction (qRT-PCR) analysis of *H19* (**A**) and miR-675 (**B**) performed on RNA extracted from human non-alcoholic fatty liver disease (NAFLD) liver (NNL) and human cirrhotic liver (cirrhosis) (*n* = 7). (**C**) Expression level of miR-675 in healthy (FN), non-tumorous (FNT), and tumorous (malignant and benign) human liver samples. (**D**) Correlation between *H19* and miR-675 expression levels in human benign and malignant liver tumors. (**E**) *H19*/miR-675 correlation in RNA samples extracted from liver tumors of Mdr2-KO mice ((Female) Fn = 9; (Male) Mn = 8)). (**F**) *H19*/miR-675 correlation in combined Mdr2-KO mice liver RNA samples, which include non-tumor (N) and tumor (T) liver samples, which arise spontaneously (Spon) (Fn = 4; Mn = 4), or following partial hepatectomy (Phx) (Fn = 6; Mn = 4), or liver samples taken from sham operated mice (Sham) (Fn = 4; Mn = 4) and from Mdr2-heterozygous mice (untreated N) (Fn =4; Mn = 6). RNA was isolated and analyzed by qRT-PCR. *H19* levels were normalized to HPRT, and miR-675 levels were normalized to RNU48. Expression correlation was calculated using Spearman’s correlation coefficient. Error bars = SD. * *p* < 0.05; ** *p*  <  0.01; *** *p* < 0.001; **** *p* < 0.0001.

miR-675 is processed out of the first exon of *H19* [13], and it has been previously reported that *H19* and miR-675 expression levels are positively correlated in various cancerous tissues, including gastric cancer, glioma, and colorectal cancer [18,19,20]. *H19* levels were shown to have increased in fibrotic and cirrhotic livers [12]. We therefore tested the levels of *H19* and miR-675 expression in human cirrhotic livers and compared it with non-alcoholic fatty liver disease (NAFLD) livers. As seen in Figure 1A,B, *H19* and miR-675 levels were higher in cirrhotic livers.

We further tested the expression level of miR-675 in human HCC samples (patient clinical characteristics shown in Table S1) and compared it to that in healthy, non-tumorous and in hepatic adenomas samples. As seen in Figure 1C, miR-675 expression level in the majority of tumors was lower than in healthy or non-tumor liver tissues. Most malignant tumors exhibited lower miR-675 expression compared to benign tumors. We next analyzed the correlation between *H19* and miR-675 within human benign and malignant liver tumors samples and found a significant positive correlation between their expression levels (Figure 1D) (Spearman’s *R* = 0.62). Similar positive *H19*/miR-675 correlation was also observed when malignant liver tumors were tested separately (Figure S1). Interestingly, two distinct groups appeared, in accordance with the miR-675 expression pattern that was observed for all liver tumors (Figure 1C).

The human non-tumorous and tumorous samples were derived from patients with a chronically inflamed liver. We therefore tested the correlation between *H19* and miR-675 expression levels in the livers of Mdr2-knockout mice (Mdr2-KO), a model of chronic inflammation-mediated HCC [21] that we investigate for many years [22,23,24]. Increased *H19* expression in tumorous and non-tumorous liver tissues of these mice has previously been shown by us [22]. We tested *H19* and miR-675 levels in RNA extracted from tumorous and non-tumorous liver tissues of male and female Mdr2-KO mice, from Mdr2-KO mice after partial hepatectomy or sham operated mice, and from Mdr2-heterozygous mice. Spearman’s *R* coefficient was calculated either separately for tumor and non-tumor samples or for all liver tissue samples combined. Similar to human HCC samples, mouse HCC samples exhibited a highly significant positive correlation between *H19* and miR-675 levels (Figure 1E; Spearman’s *R* = 0.52). This positive correlation was significant also when all tested mouse subsets were combined (Figure 1F; Spearman’s *R* = 0.34).

### 2.2. miR-675 Inhibits Cell Growth of Cultured Human HCC-Derived Cells

**Figure 2:**
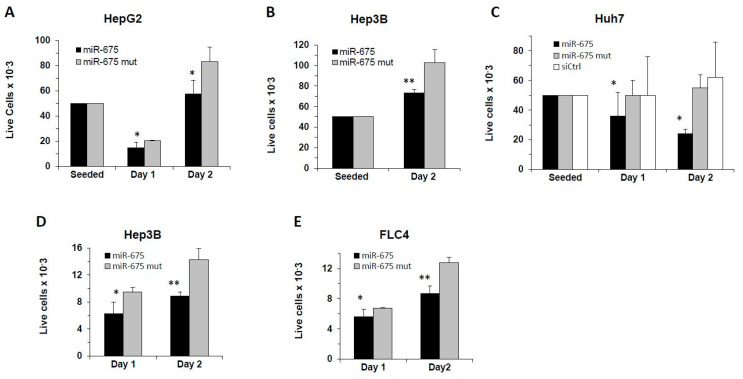
miR-675 inhibits cell growth of human HCC-derived cell lines. Cells were transfected with miR-675, with mutated miR-675 (miR-675 mut) or control Luciferase siRNA (siCtrl). Next day, cells were counted and seeded at sub-confluency with equal numbers. At the indicated time points after seeding, live cells were counted in the presence of trypan blue: (**A)** HepG2, (**B**) Hep3B, and (**C**) Huh7; or analyzed by flow cytometric analysis (FACS) following propidium iodide (PI) staining: (**D**) Hep3B and (**E**) FLC4. (*n* = 3). Error bars = SD. * *p* < 0.05; ** *p*  < 0.01.

There are conflicting data in the literature regarding the effect of *H19* and miR-675 on cell growth and, more specifically, whether it promotes or inhibits cancer cell growth and proliferation. To test the effect of miR-675 on hepatic cell line growth, we transfected human HCC-derived cell lines (HepG2, Hep3B, Huh7, and FLC4) with miR-675 or control miRs (mutated miR-675 or siLuc; see Table S3), and followed cell viability by counting live cells using trypan blue dye at several time points after seeding (Figure 2A–C) or by flow cytometric analysis (FACS) analysis following propidium iodide (PI) staining (Figure 2D,E). In all the tested cell lines, live cell number was significantly reduced by day 2 post-transfection compared to the mutant miR-675 control. The inhibition of cancer cell growth by miR-675 is in line with the data shown in the previous section—that is, miR-675 expression level in the majority of tumors was lower than in healthy or non-tumor liver tissues.

### 2.3. Negative Correlation between Fas-Associated Protein with Death Domain (FADD) and miR-675 in Human and Mouse HCC

**Figure 3:**
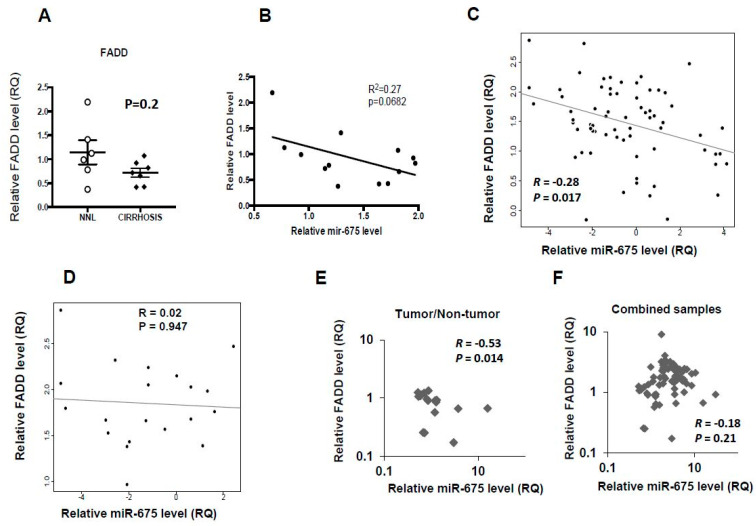
Negative miR-675/FADD (Fas-Associated Protein with Death Domain) correlation in liver samples from human and Mdr2-KO mice. (**A**) qRT-PCR analysis of FADD performed on RNA extracted from human non-NAFLD liver (NNL) and human cirrhotic liver (cirrhosis). (**B**–**F**) miR-675/FADD correlation in (**B**) in human cirrhotic liver samples; (**C**) combined human samples taken from normal and cirrhotic adult liver, fetal liver, and non-tumor and tumor samples of hepatic adenoma and HCC; (**D**) human HCC samples only; (**E**) RNA samples extracted from tumor and non-tumor liver tissues of Mdr2-KO mice (as in Figure 1E); and (**F**) combined Mdr2-KO mice liver RNA samples (as in Figure 1F). RNA was isolated and analyzed by qRT-PCR. FADD levels were normalized to HPRT, and miR-675 levels were normalized to RNU48. Expression correlation was calculated using Spearman’s correlation coefficient.

We next compared putative miR-675 target genes from TargetScan database with upregulated genes from a previously published gene array set [25] (Table S2). We chose to focus on FADD, an adaptor gene that mediates cell death and apoptotic signals, which was previously shown to be targeted by miR-675 [26]. Based on these findings, we determined FADD levels in human cirrhotic livers and compared them to those in non-NAFLD livers. Figure 3A demonstrates that FADD levels were lower in human cirrhotic livers, although not statistically significant. A negative miR-675/FADD correlation also exists in these human cirrhotic liver samples (Figure 3B). We then examined the correlation between miR-675 and FADD in combined human liver RNA samples, which included normal and cirrhotic adult liver, fetal liver, and non-tumor and tumor samples of hepatic adenoma and HCC. In these combined samples, we found a negative correlation between the expression of miR-675 and FADD (Figure 3C; Spearman’s *R* = −0.28). However, no correlation was found when HCC samples were analyzed separately (Figure 3D).

We then compared miR-675 to FADD transcript levels in livers of Mdr2-KO mice. We found a significant negative correlation in the ratio between tumorous and non-tumorous tissues (Figure 3E), but not when all tested mouse subsets were combined (Figure 3F).

### 2.4. Regulation of FADD by miR-675 In Vitro and In Vivo

**Figure 4:**
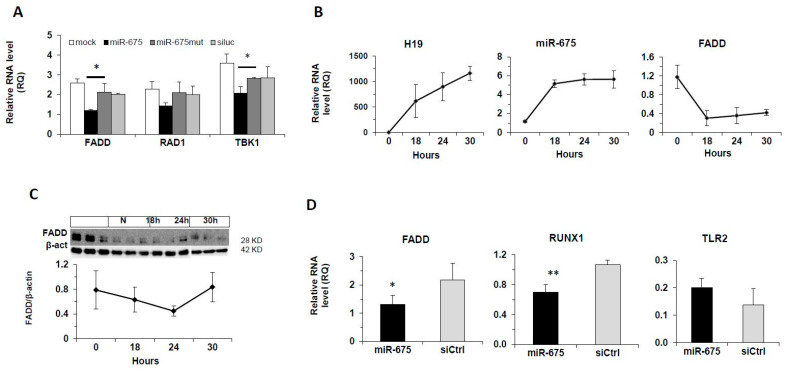
Regulation of FADD by miR-675 in vitro and in vivo. (**A**) The effect of miR-675 on target gene expression in Hep3B cells. qRT-PCR on RNA extracted from Hep3B cells 48 h after transfection with miR-675 or controls (mutated miR-675 and siLuc) (50 nM). (**B**,**C**) Hypoxia upregulates *H19* and miR-675 and downregulates FADD expression in vitro. FLC4 cells were incubated under hypoxic conditions (0.1% O_2_) for the indicated times. At each time point, RNA and proteins were extracted. (**B**) qRT-PCR for *H19*, miR-675, and FADD (*n* = 3). (**C**) Western blot analysis of FADD protein expression (top, scanned images of blot; bottom, densitometric quantification). (**D**) miR-675 overexpression downregulates FADD in mice. Balb/C mice were tail-vein-injected with miR-675 (5 μg) or control (siCtrl) and liver RNA extracted 24 h later (*n* = 3). qRT-PCR was performed for FADD, RUNX1 (positive control), and TLR2 (negative control). mRNA levels were normalized to HPRT, and miR-675 levels were normalized to RNU-6. Error bars = SD. * *p* < 0.05; ** *p*  < 0.01. RAD1 = a DNA damage repair complex protein; TBK1 = TANK Binding Kinase 1; RUNX1 = Runt-Related Transcription Factor 1; TLR2 = Toll Like Receptor 2.

To investigate the effect of miR-675 on FADD level in our experimental system, we transfected Hep3B cells with miR-675 and measured the mRNA level of FADD and the FADD target genes RAD1 and TBK1 (a protein in the non-canonic NF-kB pathway). As seen in Figure 4A, FADD and the FADD target gene TBK1 were significantly downregulated by miR-675, while another FADD target gene, RAD1 (a DNA damage repair complex protein), was reduced but not significant statistically.

We have previously shown that under hypoxic conditions the expression of both *H19* and miR-675 is induced [27]. As expected, exposing FLC4 cells to hypoxic conditions strongly induced *H19* (>1000 fold) as well as miR-675, which was also induced almost 6-fold (Figure 4B), although to a much lesser extent than *H19*. It has been shown that generation of miR-675 from *H19* is a regulated process and in most cases the abundance of miR-675 is much lower than *H19* [28]. In accordance with the increase in miR-675, FADD mRNA expression and protein abundance were downregulated (Figure 4B,C).

To explore the regulation of FADD by miR-675 in vivo, we tail-vein-injected BALB/C mice with miR-675 (an administration protocol that enables liver targeting [29]). Twenty-four hours after injection, the mice were sacrificed and liver RNA was extracted to determine the expression levels of miR-675 and FADD. In response to miR-675 injection, the liver FADD level reduced, as well as RUNX1 (a miR-675 target gene) [30], while the level of TLR2, the negative control target gene, did not reduce (Figure 4D).

### 2.5. Overexpression of miR-675 Affects the Inflammatory Response in Mice

**Figure 5:**
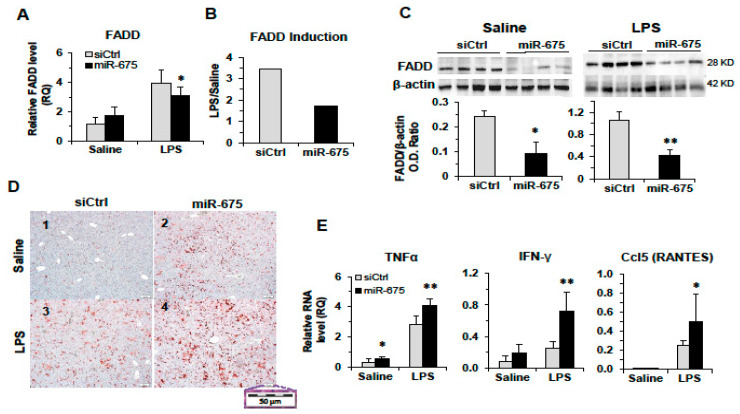
The effect of miR-675 on the inflammatory response in mice. Balb/C mice were tail-vein-injected with miR-675 or control miR (siCtrl) (5 µg), and 24 h later, the mice were injected intraperitoneal (IP) with LPS (25 µg) or saline. Liver RNA and proteins were extracted 24 h after IP injections (*n* = 5). (**A**) qRT-PCR performed for FADD RNA. (**B**) Fold induction of FADD RNA calculated by dividing the qRT-PCR result for LPS with the result for saline. (**C**) Western blot analysis of FADD protein expression following saline or LPS treatment (top, scanned images of blot; bottom, densitometric quantification). (**D**) miR-675 promotes macrophage infiltration to the liver. Representative images of f4/80 immunohistochemical macrophage staining of liver sections (magnification ×20). (**E**) qRT-PCR analysis of liver RNA for Tumour Necrosis Factor Alpha (TNFα), Interferon Gamma (IFNγ), and Regulated on Activation, Normal T cell Expressed and Secreted (RANTES). mRNA levels were normalized to HPRT. Error bars = SD. * *p* < 0.05; ** *p*  < 0.01.

Extensive studies for many years have revealed an intimate connection between cell death and inflammation [31] as well as the link between inflammation, fibrosis, and hepatocellular carcinoma [32]. The protein encoded by the FADD gene is an adaptor molecule that interacts with members of the tumor necrosis factor receptor superfamily and mediates cell death and apoptotic signals. We were interested in studying the effect of miR-675 on FADD during liver inflammation. We injected mice with miR-675 or control miR, and 24 h later, the mice were injected with lipopolysaccharide (LPS) to induce acute inflammation. Twenty-four hours after LPS injection, the mice were sacrificed and the livers were taken for RNA and protein extraction as well as for histology. In the LPS-injected mice, addition of miR-675 caused a decrease in FADD RNA expression compared to control siRNA (Figure 5A), reducing the induction rate of FADD transcript by LPS twofold (Figure 5B). Analysis of FADD protein expression level also revealed a significant decrease in miR-675-treated mice compared to control in both LPS-treated mice and saline-injected mice (Figure 5C).

To further explore the effect of miR-675 on inflammation, we performed a histological analysis of the liver sections taken from the mice treated with miR-675 followed by LPS injection (as described in the previous section). Specific immunostaining revealed a significant increase in macrophage infiltration to the liver as a result of miR-675 injection, compared to control miR, without induction of inflammation (Figure 5A panels 1 and 2). Macrophage infiltration was significantly enhanced when miR-675 and LPS treatments were combined (Figure 5A, panels 3 and 4).

It is well established that LPS promotes Tumour Necrosis Factor Alpha (TNFα) production in the liver, which in turn activates NF-κB [33]. In the presence of miR-675, we see an increase in TNFα expression in the liver following LPS treatment but also without the trigger of inflammation (Figure 5E). To test for NF-κB activation in the livers in response to miR-675 injection, we examined the expression of two pro-inflammatory cytokines, Interferon Gamma (IFNγ) and Ccl5 (RANTES, Regulated on Activation, Normal T cell Expressed and Secreted), which are known to be induced by NF-κB [34]. As seen in Figure 5E, in the presence of miR-675, following LPS treatment, the expression of IFNγ and RANTES was significantly higher in the miR-675-receiving mice compared to control miR. These results suggest that miR-675 has an effect on the inflammatory response in the liver, skewing it toward the direction of enhanced inflammation.

### 2.6. miR-675 Promotes Necrosis in Cultured Cells and Murine Livers

**Figure 6:**
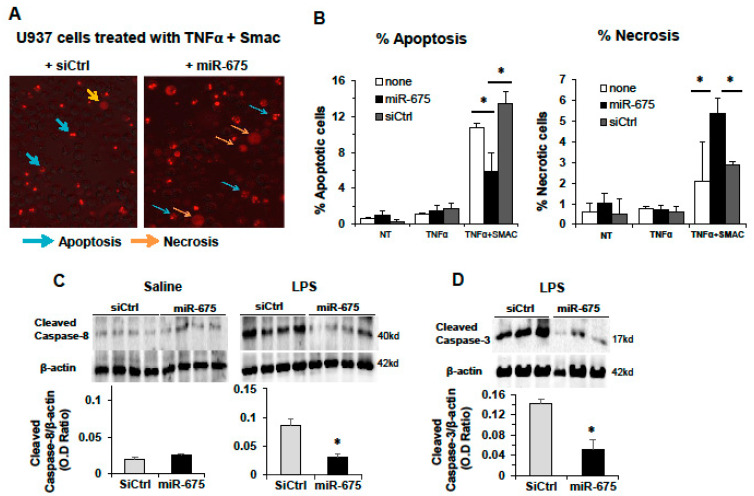
miR-675 induces necrosis in vitro and inhibits apoptosis in livers of LPS-treated mice. (**A**,**B**) miR-675 induces necrosis in vitro in U937 cells (human monocyte cell line). U937 cells were transfected with miR-675 or control siRNA (siCtrl) (50 nM) or untreated (NT). Next day, the cells were seeded with PI (1 μg/mL) in the media and treated with TNFα (100 ng/mL), TNFα+Smac (20 nM), or untreated. (**A**) Microscopy images taken 6 h after treatment using the EVOS microscope imaging system. (**B**) PI-positive cells were counted and separated from apoptotic or necrotic cells according to nuclear morphology and plotted as percentage of total cells counted in the field (live and dead). (**C**,**D**) miR-675 inhibits apoptosis in mice. Balb/C mice were tail-vein-injected with miR-675 or miR control (siCtrl) (5 µg), and 24 h later, they were injected IP with LPS (25 µg) or saline. The livers were extracted 24 h after LPS injection (*n* = 5). (**C**) Western blot analysis of cleaved caspase-8 and (**D**) cleaved caspase-3. * *p* < 0.05.

We sought to further characterize the cell death phenotype and inflammation induced by miR-675 in an additional in vitro model. The human monocyte-derived U937 cell line allows for discrimination between apoptotic and necrotic cell death in vitro, by observing the PI nuclear staining patterns of dying cells. The DNA of cells undergoing apoptosis is condensed, appearing as many small, stained foci in the nucleus, while necrotic cell DNA remains “relaxed” and PI staining is diffused throughout the nucleus or the cell (after nuclear membrane integrity is compromised) [35] ( Figure 6A). We transfected U937 cells with either miR-675 or control miR, then stained the cells with PI and induced cell death the next day using TNFα alone or TNFα in combination with Smac, to specifically induce apoptosis (Smac is a mitochondrial activator of caspases [36]). Quantitative analysis of dead cells according to morphology (Figure 6B) clearly demonstrates that in cells treated with TNFα in combination with Smac and transfected with control miR, apoptosis was the main cause of cell death (13.5% apoptotic, 2.8% necrotic cells). In contrast to control miR, in miR-675 transfected cells, apoptosis was reduced about 2-fold (from 13.5% to 6%), while necrosis was increased almost 2-fold (from 2.8% to 5.3%) (Figure 6B). These results demonstrate that transfection with miR-675 significantly increases necrotic and reduces apoptotic cell death.

To further investigate the effect of miR-675 on apoptotic or necrotic cell signaling pathways, we analyzed the level of cleaved caspase-8 (the activated form of caspase-8 and apoptosis). We demonstrate that administration of miR-675 to LPS-treated mice significantly reduced cleaved caspase-8 protein abundance compared to LPS-treated mice or to mice treated with saline alone (Figure 6C). To further strengthen these results, we also determined the level of cleaved caspase-3 (the executioner caspase of apoptosis). As seen in Figure 6D, cleaved caspase-3 protein abundance was also reduced following miR-675 administration in LPS-treated mice compared to control siRNA. These results strongly suggest that miR-675 does not activate the apoptotic cellular pathway but rather promotes a distinct cell signaling pathway of necrosis.

### 2.7. miR-675 Induces Necroptosis in a Human HCC Cell Line and in Mice

**Figure 7:**
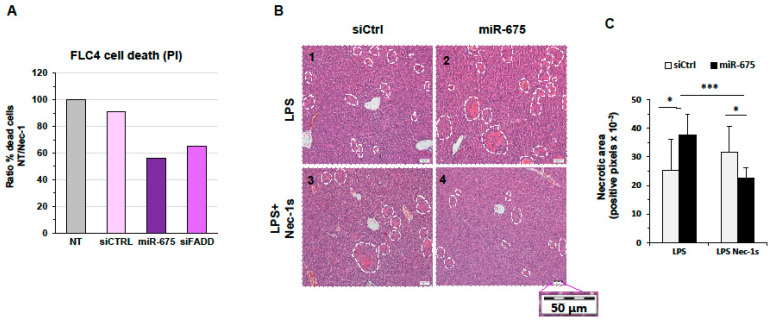
miR-675 induces necroptosis, which is rescued by Nec-1 in vitro and in vivo. (**A**) miR-675 promotes necroptosis in FLC4 cells. The cells were transfected with miR-675 or siCtrl (a negative control) or siFADD as a positive control (50 nM) together with Nec-1 (100 nM), which was administered twice, 1 h before and 24 h after transfection. The percentage of dead cells was determined 48 h after transfection by PI staining and FACS analysis. (**B**,**C**) miR-675 promotes necroptosis in mice livers. (**B**) Representative ×20 images of H & E-stained liver sections of Balb/C mice that were tail-vein-injected with miR-675 or control miR (siCtrl) (5 µg) and then injected IP with Nec-1s (125 µg Nec-1s in 200 µL saline per mouse); 24 h later, the mice were injected IP with LPS (25 µg) in addition to Nec-1s or saline. The livers were extracted 24 h after LPS injection (*n* = 5). (**C**) Quantification of necrotic areas in liver slides. Error bars = SD. * *p* < 0.05, *** *p* < 0.001.

Since morphologically it is difficult to differentiate between necrosis and necroptosis, specific necroptosis inhibitors are used to characterize necrotic cell death pathway, e.g., the RIP1 inhibitor necrostatin-1 (Nec-1) [37]. We first wanted to analyze the nature of cell death induced by miR-675 in cultured HCC-derived cells. We treated FLC4 cells with Nec-1 one hour prior to transfecting the cells with either miR-675 or controls. The following day, we treated the cells again with Nec-1 (since it has a short half-life). The next day (48 h after transfection), the cells were stained with PI and counted using FACS analysis. Figure 7A shows the ratio of dead cells between nontreated and Nec-1 treated cells. Significantly, more FLC4 cells died in the presence of miR-675 and, similarly, in the presence of siFADD compared to the negative control (Figure 7A).

We next wanted to determine whether the necrosis we observed in the mice injected with miR-675 is necroptosis. To test this, BALB/C mice were tail-vein-injected with miR-675 and, 24 h later, injected IP with LPS, as described in the previous section. In addition, half of the mice were injected IP twice with Nec-1s (a Nec-1 derivative [37]) or saline as control—once immediately after miR-675 injection and again 1 h after LPS treatment. The following day, the mice were sacrificed and the livers were collected. Hematoxylin and Eosin (H & E) staining was performed, and the color thresholding method was used to quantify necrotic areas (Figure 7B,C). Our results demonstrate that the presence of miR-675 significantly augments the necrotic damage that occurs in the livers following LPS treatment (Figure 7B panel 1 compared to 2 and Figure 7C). In the miR-675-injected livers, but not in control mice, necrosis was inhibited to a large extent by administration of Nec-1s (Figure 7B panel 3 compared to 4 and Figure 7C). Since Nec-1s inhibits necroptosis and not nonregulated-necrosis or apoptosis, our results indicate that miR-675 promotes necroptosis in vivo.

### 2.8. miR-675 Promotes Necroptosis by Enhancing Mixed Lineage Kinase Domain-Like Pseudokinase (MLKL) Phosphorylation

**Figure 8:**
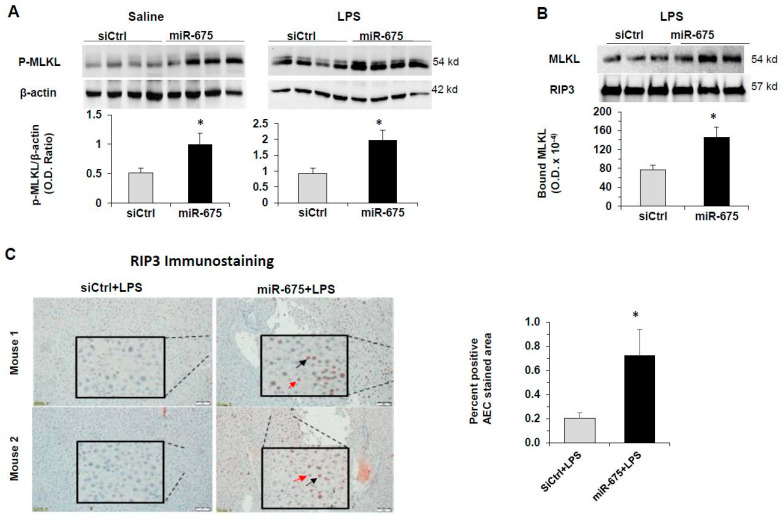
miR-675 increases phospho-MLKL protein levels, the level of MLKL bound to RIP3 and nuclear RIP3 in the mouse liver. Balb/C mice were tail-vein-injected with miR-675 or miR control (siCtrl) (5 µg), and 24 h later, they were injected IP with LPS (25 µg) or saline. The livers were collected 24 h after IP injections. (**A**) Western blot analysis of phospho-MLKL (p-MLKL) in proteins extracted from the mice livers. The graph depicts the average p-MLKL/β-actin densitometric ratios obtained for each group of mice (*n* = 5). (**B**) Western blot analysis of MLKL co-immunoprecipitated following immunoprecipitation of RIP3 from liver homogenates. The detected levels of RIP3 served as loading control for the immunoprecipitated pellets. The graph shows the densitometry of bound MLKL (*n* = 3). (**C**) RIP3 immunostaining of liver sections of the mice. Representative ×10 images of RIP3-stained liver sections. Left panels: images of siCtrl+LPS-treated mice. Right panels: miR-675 + LPS-treated mice. The images represent two different mice from each treatment. Black arrow indicates positive RIP3 in liver nuclei. The red arrow indicates positive RIP3 in liver immune cells. The graph depicts the average of the counted positive 3-Amino-9-Ethylcarbazole (AEC)-stained area out of total area (7 fields per slide were counted) (*n* = 3). Error bars = SD. * *p* < 0.05.

An important trigger for necroptosis is the phosphorylation of mixed-lineage kinase domain-like (MLKL) protein by RIP3 [8]. Phosphorylation leads to oligomerization of MLKL, which disrupts membrane integrity and subsequently leads to cell death. To verify further that miR-675 induces necroptosis in the liver, we tested the effect of miR-675 injection into mice on the level of phosphorylated MLKL. Proteins were extracted from the livers of mice treated with miR-675 or control miR, followed by injection either with LPS or with saline as control. Western blot analysis revealed that miR-675 significantly enhanced phosphorylated MLKL (p-MLKL) levels in LPS-treated mice. Enhancement of p-MLKL was observed also in mice treated with saline, i.e., without the additional inflammatory trigger (Figure 8A).

Since it was shown that in necroptosis, RIP1 phosphorylates RIP3, which, in turn, phosphorylates MLKL [7], we wanted to learn whether miR-675 treatment of mice affects the binding of MLKL to RIP3. We therefore performed immunoprecipitation of RIP3 from liver homogenates of the mice treated with LPS and miR-675 and measured, by Western blot analysis, the level of MLKL protein that was co-immunoprecipitated with RIP3. As seen in Figure 8B, following miR-675 treatment, significantly more MLKL was bound to RIP3 compared to control.

To further confirm our hypothesis that miR-675 induces necroptosis in liver hepatocytes, we performed RIP3 immunostaining in sections obtained from the mice injected with miR-675 or control miR and further injected with LPS. Our data demonstrate that following treatment with LPS, a higher abundance of RIP3 is found within the hepatocytes of the mice treated with miR-675 compared to the mice treated with control miR (Figure 8C,D). Taken together, these results indicate that miR-675 can promote necroptosis in the livers of treated mice through the RIP3-MLKL pathway.

## 3. Discussion

*H19* and miR-675 have been shown to be elevated in many cancers, including inflammation-induced HCC, but their roles in cancer are controversial, with some researchers suggesting tumor suppressor activity and others demonstrating pro-tumorigenic activity [11,38,39]. In this study, we found that transfection of miR-675 inhibited the growth of HCC-derived cell lines and promoted cell death. We surveyed databases and found FADD to be a putative candidate to link miR-675 and cell death. We validated that FADD is downregulated by miR-675 both in vitro and in vivo. Accordingly, we found a negative correlation between miR-675 and FADD in combined human liver RNA samples, which included normal and cirrhotic adult liver, fetal liver, and non-tumor and tumor samples of hepatic adenoma and HCC. A negative FADD/miR-675 correlation was also found in human cirrhotic liver samples that were analyzed separately (Figure 3B) but was not found in HCC tumors analyzed separately (Figure 3D). We speculate that this could be due to the reduced number of tumor cells that express *H19* and miR-675 or due to as yet unknown mechanisms that abrogate the degradation of FADD mRNA by miR-675.

A recent paper has shown that *H19* and miR-675 increase in gastric cancer, and overexpression of *H19* and miR-675 promotes cell proliferation and inhibits apoptosis by downregulation of FADD [26]. Here, we also found that hypoxia significantly induces both *H19* and miR-675 in HCC cell lines and this is followed by a miR-675-dependent downregulation of FADD, and that miR-675 inhibits apoptosis. Thus, we characterized a cascade in which hypoxia elevates *H19* and miR-675, which in turn downregulates FADD. A cycle of hypoxia and inflammation has been described in many tissues, and it is known that hypoxia can trigger the inflammatory response and severe inflammation involves hypoxic conditions. Hypoxia is an essential step in tumor growth, and it develops within the growing tumor [40,41].

FADD is a critical adaptor protein for death receptor-mediated apoptosis, but when apoptosis is attenuated, due to inhibition of signaling components including caspase 8, necroptosis can be triggered [9,42]. Necroptosis, a regulated form of necrotic cell death, is considered to be a strong trigger of inflammation and innate immune response [6]. At present, multiple pathways of regulated cell death are known to be implicated in various human pathologies [43]. When examining the livers of miR-675-injected mice, we observed significant necrotic regions in these mice compared to controls. To demonstrate that the necrosis induced by miR-675 in vivo was essentially necroptosis, we measured MLKL phosphorylation, a hallmark of necroptosis [8], and found a higher level of phosphorylated MLKL in the livers of miR-675-injected mice, indicating an increase in necroptosis induction. Furthermore, we found that cleaved caspase-8 and cleaved caspase-3 were inhibited following miR-675 administration. In addition, a higher level of MLKL protein was co-immunoprecipitated with RIP3, following treatment of mice with miR-675. In previously suggested models for the nuclear events leading to necroptosis induction [8,44], nuclear RIP1 serves as a scaffold for nuclear RIP3 kinase activation. RIP3 is then phosphorylated and associates with MLKL, which is further phosphorylated, and subsequently, the phosphorylated MLKL translocates to the plasma membrane and causes its permeabilization, leading to necroptosis. In accordance, following RIP3 immunostaining of liver sections obtained from mice treated with miR-675 in combination with LPS, we found a high abundance of nuclear RIP3 within the hepatocytes. In addition, RIP3 was also shown previously to be expressed in immune cells within the liver, and our data support this as well.

**Figure 9:**
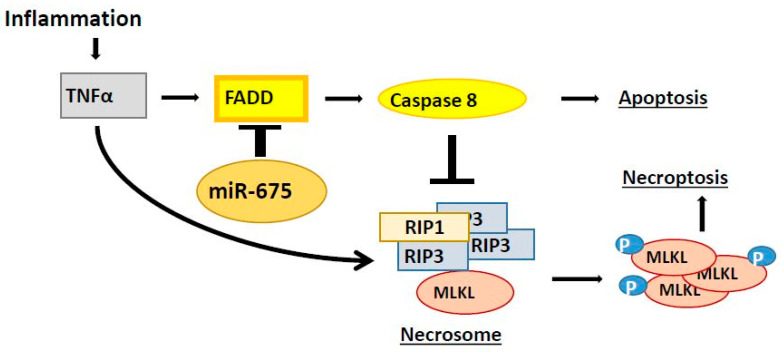
Our schematic model for induction of necroptosis by miR-675. Necroptosis occurs following the rupture of the cell membrane, which involves phosphorylated MLKL molecules. Phosporylation of MLKL requires prior assembly of the necrosome, which contains RIP1 and RIP3 molecules. Caspase-8, which is activated by FADD, induces apoptosis and inhibits necrosome formation due to cleavage of RIP1 and RIP3. Administration of miR-675, which targets the FADD gene, leads to inhibition of caspase-8 activation, thus enabling the assembly of the necrosome, resulting in necroptosis. TNFα = Tumour Necrosis Factor Alpha; RIP = kinase receptor-interacting proteins; MLKL = Mixed Lineage Kinase Domain-Like Pseudokinase.

We further confirmed our finding of necroptosis induction by miR-675 using the necroptosis inhibitor Nec-1, which rescued miR-675 transfected cells from dying. We also showed that Nec-1 significantly reduces the extent of necrosis in the livers of miR-675- and LPS-injected mice. Taken together, these results demonstrate that the cell death induced by miR-675 is due to necroptosis, as we suggest in our model posted in Figure 9.

The net effect of necroptosis on cancer progression remains unclear. On the one hand, necroptosis contributes to cancer cell death and inhibits tumor initiation and progression, by killing the tumor cells. On the other hand, cell death also increases the risk of proliferation and metastasis of the surviving cells by inducing reactive oxygen species, activation of inflammation, and suppression of the immune response [6,45].

Previous research shows that *H19* is able to enhance NF-κB activation, thus contributing to cell survival and proliferation [46]. In this work, we demonstrate that this function of *H19* can be attributed, at least partially, to miR-675 activity. We show that miR-675 treatment enhances in vivo several NF-κB target genes, specifically pro-inflammatory cytokines. We also see macrophage recruitment in the livers of miR-675-injected mice, and this is further amplified by the administration of LPS. These results strongly suggest that miR-675 enhances liver inflammation via increased activation of NF-κB.

Taken together, our results demonstrate that miR-675 promotes necroptosis in tumor cells and also intensifies liver inflammation, both of which may possess an anti-tumorigenic effect.

Remarkably, in most human HCCs, the miR-675 level is low. In those tumors where the miR-675 level is higher, no negative miR-675/FADD correlation was observed (Figure 3D). This may suggest that in human HCCs the pro-necroptotic activity of miR-675 is abrogated either by decreasing its level or by disrupting its effect on FADD by an as yet unknown mechanism. Thus, modulation of miR-675 levels may lead to new therapeutic approaches.

## 4. Materials and Methods

### 4.1. Human Samples

All human samples were derived from surgical specimens of humans undergoing liver surgeries. These included tumorous and non-tumorous tissues. Normal human samples were derived from human liver tissue without liver parenchymal pathology. Benign tumors were derived from hepatectomy for liver adenomas, and malignant tumors were human HCC. All samples were collected following the signing of an informed consent by patients under institutional and national regulations.

The study of patients (shown in Table S1) was approved by the local Ethics committee (CCPPRB Paris Saint-Louis IRB00003835). The use of the Human Liver Biobank and the study protocol (shown in Table S6) were approved by the Research Ethics Board of the McGill University Health Centre (MUHC).

### 4.2. Animals and Animal Procedures

Mice were handled according to the criteria outlined in the Guide for the Care and Use of Laboratory Animals, prepared by the National Academy of Sciences and published by the National Institutes of Health, and the Hebrew University-Hadassah Medical School Ethics Review Board (Ethical No. MD-14-14131-3). Eight-week-old BALB/C mice were purchased from ENVIGO (Jerusalem, Israel). The wt FVB and FVB.129P2-Abcb4tm1Bor (Mdr2-KO/FVB) mice were purchased from the Jackson Laboratory (Bar Harbor, ME, USA) (. Heterozygote mice were created by breeding with wt FVB mice (described in [22]). For correlation analysis, 70% partial hepatectomy (PHx) or sham surgery was performed [23] on 6-month-old and 9-month-old (preneoplastic stages) Mdr2-KO/FVB mice. For spontaneous tumor analysis, 13–14-month-old Mdr2KO mice were sacrificed. Hydrodynamic tail vein injection of BALB/C mice was performed with 1.8 mL saline or with saline containing 5 µg of miR-675 or siCtrl (scrambled miR). Liver non-tumor and tumor tissues extracted from the treated mice were frozen in liquid nitrogen and kept at −80 °C for RNA and protein analyses, or formalin fixed for histological analysis.

### 4.3. Cell Lines and Cell Culture

HEK293 cells and the HCC-derived human cell lines—Huh7, HepG2, Hep3B, and FLC4—were cultured in DMEM. The oncogenic human monocyte cell line U937 was cultured in RPMI. Both media were supplemented with 10% fetal calf serum (FCS) and 1% penicillin/streptomycin. Exposing cell cultures to hypoxic conditions (20% CO_2_, and less than 0.1% O_2_) was achieved using an anaerobic jar equipped with AnaeroPack (Mitsubishi Gas Chemical, Tokyo, Japan).

### 4.4. Plasmids

To construct the plasmid carrying the 3′-UTR region of the human FADD gene (810 bp), containing the putative miR-675 target sites, this 3′-UTR region was polymerase chain reaction (PCR)-amplified from human liver DNA using primers P584 and P585 (see Table S4). The PCR fragments were inserted into the pmirGLO vector (Promega Corporation, Madison, WI, USA) between the NheI and XbaI restriction sites, downstream of the firefly luciferase reporter gene. The plasmid with the mutated miR-675 recognition sequence in the 3′-UTR of FADD was generated from the plasmid carrying the wt FADD 3’-UTR, using primerP1114. The pmirGLO vector carrying the 3′- UTR of the human DNMT1 was previously constructed and described by Rivkin et al. [47].

### 4.5. Transfection Procedures and Luciferase Activity Assay

Transfection of cells was performed with Lipofectamine 2000 transfection reagent (Invitrogen, Carlsbad, CA, USA) according to the manufacturer’s protocol. For luciferase activity assay, the cells were collected 24 or 48 h following transfections, and assayed using the dual-luciferase assay System (Promega Corporation, Madison, WI, USA) on a luminometer (Mithras 2000, Centro XZ, LB960, Berthold Technologies, Bad Wildbad, Germany). Results were normalized to the Renilla luciferase control.

### 4.6. RNA Extraction and Real-Time Polymerase Chain Reaction (PCR)

Total RNA from cell lines and mice tissues was isolated using Trizol reagent (Invitrogen, Carlsbad, CA USA). RNA was treated with DNaseI using the DNaseI Kit (Ambion, Ambion Inc., Austin, TX, USA). cDNA was synthesized from 0.5–1 μg total RNA using the Quanta Biosciences qScript ™ (Quanta Biosciences Inc., Gaithersburg, MD, USA) cDNA Synthesis Kit (95047-100) for mRNA analysis and the qScript™ microRNA cDNA Synthesis Kit (95107-100) for miRNA analysis. Quantiative PCR (qPCR) of miRNAs and mRNAs in RNA isolated from cultured cell or mice livers was performed using the CFX384, C1000 touch thermal cycler (Bio-Rad, Hercoles, CA, USA) and a SYBR Green PCR Kit: Quanta Cat. #84018 and #84071, respectively. All experiments were performed in triplicate. qRT-PCR of RNA isolated from human samples was performed using the following specific TaqMan (TAqMan, Invitrogen, CA, USA) predesigned probes: Hs00399294_g1 (*H19*),Hs04187499_m1 (FADD), and hsa-miR-675-002005 (miR-675).

### 4.7. Immunohistochemistry

The livers were fixed in 4% formaldehyde, dehydrated, embedded in paraffin, and sectioned (5 μm thick). For immunohistochemical analysis, the samples were incubated overnight at 4 °C with rat anti-mouse F4/80 monoclonal antibodies (AbD Serotc, Oxon, UK) at concentrations of 1:500, or anti-RIP3, followed by a secondary antibody anti-rat for mouse tissue (N-Histonefine) labeled with streptavidin horse radish peroxidase (HRP) using a 3,3′-Diaminobenzidine (DAB) staining kit (Invitrogen, Carlsbad, CA, USA). For RIP3K immunostaining, we used the anti-rabbit RIP3/RIP3K polyclonal antibody (Abcam, Cambridge, UK), followed by a secondary antibody, goat anti-rabbit IgG (DAKO North America Inc., Carpinteria, CA, USA). AEC chromogen was used as a signal detector (observed as the red color). An Olympus BX61microscope (Olympus, Tokyo, Japan) was used for image acquisition. For quantification of the positive-stained areas, we used Image J software-win3-2 (National Institute of Health, Bethesda, MD, USA).

### 4.8. Western Blot Analysis

Equal amounts of lysates containing 30 μg of total protein were separated on 8% or 12% SDS PAGE gels and electrotransferred onto nitrocellulose membranes (Schleicher and Schuell, Keene, NH, USA). The membranes were blocked in 1% of nonfatty milk. The membranes were incubated at 4 °C overnight with a primary rabbit polyclonal FADD antibody (1:500), primary mouse monoclonal cleaved caspase-8 (1:500), primary rabbit polyclonal cleaved caspase-3 (1:1000), or with a primary mouse monoclonal phospho-MLKL antibody (1:500), a primary MLKL antibody (1:500) (see Table S5), or a primary rabbit polyclonal RIP3/RIP3K antibody (1:500) (obtained from either Santa-Cruz Biotechnology Inc., Dallas, TX, USA, cell signaling, or Abcam Inc., Cambridge, UK). Loading control was detected using 1:15,000 β-actin with primary mouse monoclonal β-actin antibody (Abcam Inc., Cambridge, UK) (Table S5). The secondary antibodies were goat anti-mouse IgG or goat anti-rabbit IgG (DAKO North America Inc., CA, USA). Immunoblot signals were detected using enhanced chemiluminescence and quantified with scanning densitometry using Image J software-win3-2 (National Institute of Health, Bethesda, MD, USA). Protein loading and image exposure were uniform throughout all experiments.

### 4.9. Immunoprecipitation

100 μg of whole cell extracts were immunoprecipitated using a rabbit polyclonal anti-RIP3/RIP3K (Abcam Inc., Cambridge, UK), which was diluted 1:100. The samples were agitated overnight at 4 °C. Protein A/G beads (Pierce Inc., Vallejo, CA, USA) were added and the samples were agitated for two more hours at 4 °C and centrifuged at 14,000 rpm for 5 min at 4 °C. The resulting pellet was washed three times, suspended in sample buffer, and boiled for 5 min. The samples were centrifuged at 14,000 rpm for 5 min at room temperature and the resulting supernatants were subjected to immunoblotting, as described above, with mouse monoclonal anti-MLKL (Abcam Inc., Cambridge, UK) and rabbit polyclonal anti-RIP3/RIP3K (Abcam Inc., Cambridge, UK), which served as loading control.

### 4.10. Statistical Analysis

For human samples, statistical analysis and data visualization were performed using R software version 3.3.2 (R Foundation for Statistical Computing, Vienna, Austria, https://www.R-project.org). In all other experiments, statistical significance was calculated using Student’s *t*-test. Data are means ± standard deviation (SD). A one-tailed *p* < 0.05 was taken to indicate a statistically significant difference. For correlation analysis, the non-parametric Spearman’s *R* coefficient was used.

## 5. Conclusions

The long noncoding RNA *H19* is increased in most cancers as well as in chronically inflamed liver and in a subclass of highly proliferative HCC tumors. Most probably, *H19* influences hepatocarcinogenesis in multiple ways. Recently, we showed that *H19* is an oncogenic driver for HCC [45]. miR-675, which derives from the first exon of the *H19* transcript, plays an important role in the switch from apoptosis to necroptosis in HCC. The development of necroptosis enhances inflammation in the liver parenchyma. We have shown in the past that increased liver inflammation is a major driver for hepatocarcinogenesis.

In pre-tumorigenic livers, *H19* is also increased. The increase in miR-675 in this pathological state could enhance HCC development and, thus, miR-675 might become a relevant target against hepatocarcinogenesis. Further investigations are warranted toward this direction.

## Data Availability

All the data will be available upon request.

## Supplementary Materials

The following are available online at https://www.mdpi.com/2072-6694/13/3/411/s1, Table S1: Clinical characteristics of a subset of HCC patients, Table S2: List of putative miR-675 target genes, Table S3: A list of siRNAs, Table S4: A list of oligonucleotide primers, Table S5: A list of antibodies, Table S6: Clinical characteristics of patients (Figure 3A), Figure S1: miR-675/H19 correlation in human HCC samples, Whole Western Blot exposures and the specific bands exhibited in the figures of the manuscript; including Figure 5, Figure 6 and Figure 8.
