# Supplementary material for: The lncRNA H19-Derived MicroRNA-675 Promotes Liver Necroptosis by Targeting FADD
Source: Cancers (Basel). 2021 Jan 22;13(3):411. doi: 10.3390/cancers13030411 (PMC7866230; doi:10.3390/cancers13030411)
Supplement: Supplementary file 1 [file cancers-13-00411-s001.pdf]

Supplementary Materials

S1 Table. Clinical characteristics of a subset of HCC patients from Nault et al[1] selected for miR-675, H19 and FADD expression level analysis (n = 60).

Variable		Total (%)
Age (n=60) (years)	[mean, min-max]	59.5 (18-85)
Gender	Male : Female	49 (82%) : 11 (18%)
Etiology (n=60)<br>(including 9 patients<br>with ≥ 2 etiologies)	Alcohol<br>Hepatitis B<br>Hepatitis C<br>Hemochromatosis<br>Metabolic syndrome<br>Without known etiology	25 (42%)<br>20 (33%)<br>10 (17%)<br>3 (5%)<br>5 (8%)<br>7 (12%)
Tumor features (n=60)	Tumor size (mm) [mean, min-max]<br>Macro and/or micro vascular invasion	86 (16-200)<br>30 (50%)
Differentiation (n=59)	Edmonson I-II<br>Edmonson III-IV	27 (46%)<br>32 (54%)
BCLC (n=60)	[0-A]<br>[B-C]	45 (75%)<br>15 (25%)
Non-tumoral liver: Fibrosis according to METAVIR (n=60)	F0-F1<br>F2-F3<br>F4	19 (32%)<br>23 (38%)<br>18 (30%)
Preoperative serum Alpha Foeto Protein (n=57)	≤ 20 ng/ml<br>> 20 ng/ml	23 (40%)<br>34 (60%)
TERT (n=56)	M<br>NM	33 (59%)<br>23 (41%)
CTNNB1 (n=56)	M<br>NM	23 (41%)<br>33 (59%)
TP53 (n=60)	M<br>NM	15 (25%)<br>45 (75%)

[1]Nault JC1, De Reyniès A, Villanueva A, et al. A hepatocellular carcinoma 5-gene score associated with survival of patients after liver resection. Gastroenterology. 2013, 145:176-87.

The study was approved by Institutional Review Board committees (CCPRB Paris Saint Louis, 1997, 2004 and 2010). All patients gave their written informed consent according to French legislation

S2 Table. List of putative miR-675 target genes

GENE_ID	Context score as 675 target	Expression score siH19 - norm	Gene name	Pathway/ process
STARD7	-0.24	324.2	StAR-related lipid transfer (START) domain containing 7	
PHLDA1	-0.27	272.825	pleckstrin homology-like domain, family A, member 1	
**RAD1**	**-0.22**	**178.25**	**RAD1 homolog (S. pombe)**	**DNA damage repair**
ABTB2	-0.19	143.8	ankyrin repeat and BTB (POZ) domain containing 2	
**TBK1**	**-0.38**	**103**	**TANK-binding kinase 1**	**NFKB**
RXRB	-0.18	61.7	retinoid X receptor, beta	
DDX23	-0.23	53.7	DEAD (Asp-Glu-Ala-Asp) box polypeptide 23	
**FADD**	**-0.24**	**45.225**	**Fas (TNFRSF6)-associated via death domain**	**Apoptosis**
HARS	-0.22	35.425	histidyl-tRNA synthetase	
ACO1	-0.23	23.175	aconitase 1, soluble	
AP3D1	-0.24	18.6	adaptor-related protein complex 3, delta 1 subunit	
OTUB2	-0.2	16.125	OTU domain, ubiquitin aldehyde binding 2	
EPB41L2	-0.2	14.25	erythrocyte membrane protein band 4.1-like 2	
**RUNX1**	**-0.26**	**13.8**	**runt-related transcription factor 1 (acute myeloid leukemia 1; aml1 oncogene)**	**Transcription factor**
SLC6A8	-0.29	11.875	solute carrier family 6 (neurotransmitter transporter, creatine), member 8	
PKD1	-0.27	10.325	polycystic kidney disease 1 (autosomal dominant)	

Putative miR-675 target genes from TargetScan data base were cross referenced with up-regulated genes from a gene array data set taken from Matouk I, et al. PLoS One. 2007;2: 845. Genes fitting both criteria appear in this table. In bold – genes that were chosen for

S3 Table. siRNAs

Name (Species)	Sequence	Manufacturer
**miR-675-mimic (human)**	uggugcggagagggcccacagugdTdT<br>dTdTauuacgccucucccgggugucac	Sigma
**miR-675-mimic (mouse)**	uggugcggaaagggcccacagudTdT<br>dTdTaccacgccuuucccggguguca	IDT-syntezza
**miR-675-mut (human)**<br>**mutations in red**	uguucaggcgagguccgacagugdTdT<br>dTdTauaaguccgcuccaggcugucac	Sigma
**siLUC**	cuuacgcugaguacuucgadTdT<br>dTdTgaaugcgacucaugaagcu	Sigma
**siGFP**	gcugacccugaaguucaucdTdT<br>dTdTcgacugggacuucaaguag	Sigma
**siFADD (human)**	gcaguccucuuauuccuaadTdT<br>dTdTuuaggaauaagaggacugc	IDT-syntezza
**siCtrl**	caccacauaccgcacggdTdT<br>dTdTgugguguauggcgugcc	IDT-syntezza

S4 Table. Primers

Gene	Species	Direction	Sequence	Primer Number
miR-675	Human	Forward	5’-UGGUGCGGAGAGGGCCCACAGUG-3'	P1772
miR-675	Mouse	Forward	5’-UGGUGCGGAAAGGGCCCACAGU-3'	P1773
H19	Human	Forward	5’-GAGGCACTGGCCTCCAGA-3'	P1774
H19	Human	Reverse	5’-CTGGAGACCTGGCCTCGTC-3'	P1775
H19	Mouse	Forward	5' CATGTCTGGGCCTTTGAATCC-3’	P1776
H19	Mouse	Reverse	5' TTGGCTCCAGGATGATGTGG-3’	P1777
Fadd	Human	Forward	5’-CGCGTCGACGACTTCGA-3’	
Fadd	Human	Reverse	5’-CGTTAAATGCTGCACACAGGTCT-3’	
Fadd	Mouse	Forward	5’-GCGACACGATCTACTGCAG-3’	
Fadd	Mouse	Reverse	5’-TCAAATGCCACCTGCAGATCT-3’	
Fadd	Human	Forward	5'-CAAAGCTAGCGGTGGACCACAGGCATC-3'	P584
Fadd	Human	Reverse	5' -CCAAATCTAGACTGCCTTGGCAATTCTG-3'	P585
Fadd	Human	Reverse	CCCAAGCCCGGCCTCATACGTAAGATACATTA ATAAACGC-3'	P1114
Rad1	Human	Forward	5'-TGAGGAGACCCTGGACTTTG-3’	P566
Rad1	Human	Reverse	5'-GGAACTTCCTGCATTTCCAA-3’	P567
RunX1	Human	Forward	5'-CACCTACCACAGAGCCATCA-3’	P568
RunX1	Human	Reverse	5'-GCTGAGGGTTAAAGGCAGTG-3’	P569
RunX1	Mouse	Forward	5’-GGCCATGAAGAACCAGGTAGC-3’	
RunX1	Mouse	Reverse	5’-GTCAGAGTGAAGCTCTTGCCTCT-3’	
TBK1	Human	Forward	5'-GAATTTTGTCCATGTGGGAGTT-3’	P570
TBK1	Human	Reverse	5'-AGCTGCACCAAAATCTGTGA-3’	P571
GapDH	Human	Forward	5'-ACCACAGTCCATGCCATCAC-3'	P21
GapDH	Human	Reverse	5'-TCCACCACCCTGTTGCTGTA-3'	P22
TLR2	Mouse	Forward	5'-ACAACTTACCGAAACCTCAGAC-3’	P974
TLR2	Mouse	Reverse	5'-CACACACCCCAGAAGCATCACATG-3’	P975
TNFα	Mouse	Forward	5’-CTGTAGCCCACGTCGTAGCAA-3’	P1076
TNFα	Mouse	Reverse	5’-CTGGCACCACTAGTTGGTTGTCT-3’	P1077
IFNγ	Mouse	Forward	5'-CCACGGCACAGTCATTGAAAG-3'	P1029
IFNγ	Mouse	Reverse	5'-AGAGATAATCTGGCTCTGCAGG-3'	P1030
RANTES	Mouse	Forward	5'-GTGCCCACGTCAAGGAGTAT-3’	P1133
RANTES	Mouse	Reverse	5'-CTCTGGGTTGGCACACACTT-3’	P1134
HPRT	Human	Forward	5’-GGACAGGACTGAACGTCTTGC-3’	P701
HPRT	Human	Reverse	5’-CAACACTTCGTGGGGTCCTT-3’	P702
HPRT	Mouse	Forward	5'-GCGATGATGAACCAGGTTATGA-3’	P919
HPRT	Mouse	Reverse	5'-ATCTCGAGCAAGTCTTTCAGTCCT-3’	P920

S5 Table. Antibodies

Protein (Species)	Specifications	Manufacturer
**FADD (H,M)**	Rabbit polyclonal for WB	Santa Cruz (SC-5559)
**Actin-β (H,M)**	Mouse monoclonal for WB	MP biomedicals (691001)
**MLKL (H,M)**	Rabbit polyclonal for WB	Abcam (ab194699)
**P-MLKL, s345 (Mouse)**	Rabbit monoclonal for WB	Abcam (ab196436)
**F4/80 (Mouse)**	Rat monoclonal for IHC	AbD Serotc

S6 Table. Clinical characteristics of patients (Figure 3A)

Sex	Age	Type of Procedure	Diagnosis	NAS staging: Steatosis	NAS staging: Lobular Inflammation	NAS staging: Hepatocyte Ballooning	Fibrosis score	% Micro steatosis	% Macro steatosis	% Total steatosis	NA score	Grading
Female	52	Resection	Biliary cystadenoma	0	0	1	1A	0	0	0	2	LOW
Female	65	Resection	CRCLM	0	1	0	1A	3	1	4	2	LOW
Female	51	Resection	CRCLM	0	1	0	1A	0	0	0	2	LOW
Male	67	Resection	CRCLM	0	1	0	1A	1	3	4	2	LOW
Female	62	Resection	CRCLM	0	1	0	1A	0	2	2	2	LOW
Male	77	Resection	CRCLM	0	0	0	1A	0	0	0	1	LOW
Male	70	Resection	CRCLM	0	0	0	1A	0.5	0.5	1	1	LOW
Female	65	Resection	benign	0	3	1	3	0	0	0	7	cirrhotic
Male	75	Resection	benign	0	3	1 or 2	3	1	3	4	7	cirrhotic
Male	70	Resection	Klatskin tumour /Cholangiocarcinoma	0	3	2	3	0	0	0	8	cirrhotic
Male	52	Resection	Brain cancer liver met	1	2	1	2 or 3	15	10	25	6	cirrhotic
Female	45	Resection	CRCLM	2	2	2	3	28	12	40	9	cirrhotic
Female	53	Resection	Cholangiocarcinoma	0	2	2	3	0	0	0	7	cirrhotic
Male	52	Resection	CRCLM	1	2	1	3	4	12	16	7	cirrhotic

Figure S1. miR-675/H19 correlation in human HCC samples

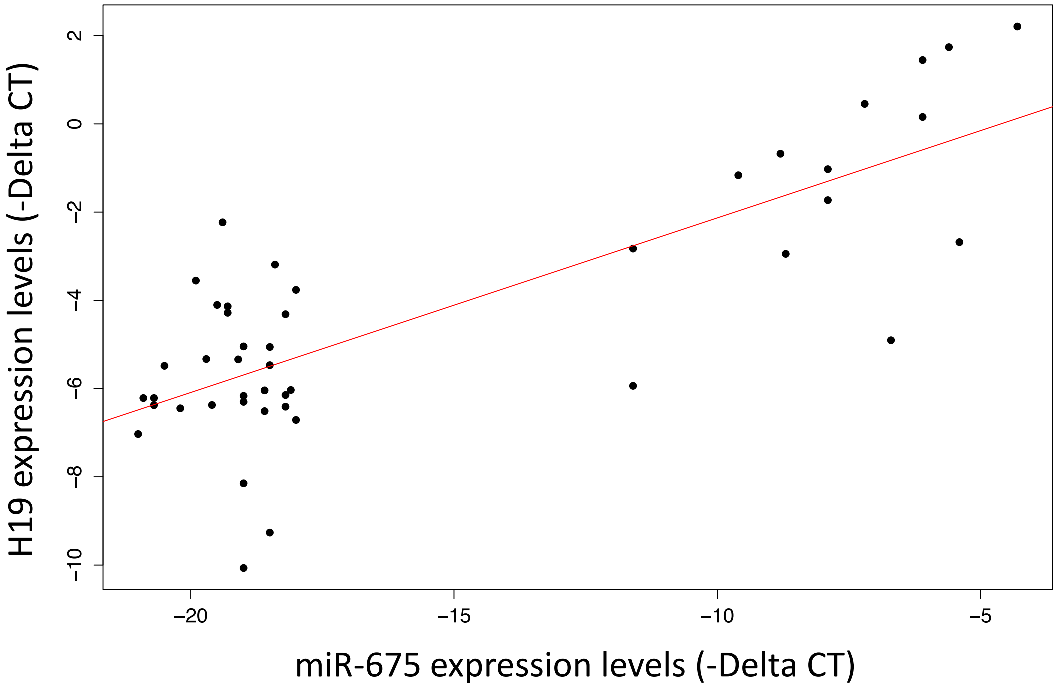

Figure S1. Correlation between H19 and miR-675 expression levels in human malignant liver tumors (taken from samples shown in Figur1C). R: 0.61, P-Value < 0.0001.

Whole Western Blots exposures and the specific bands exhibited in the figures of the manuscript; Including Figures 5, 6 and 8.

Figure 5

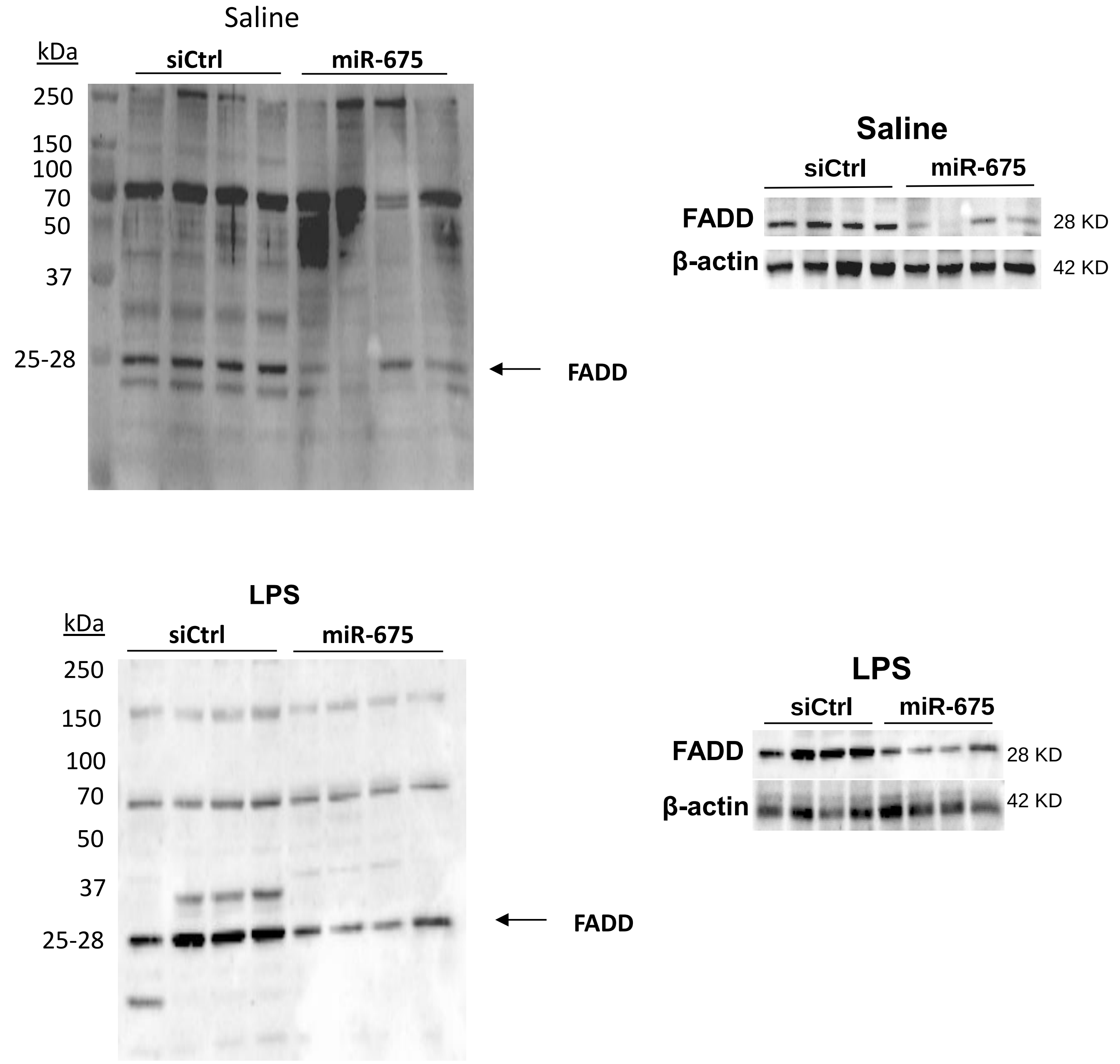

Figure 6

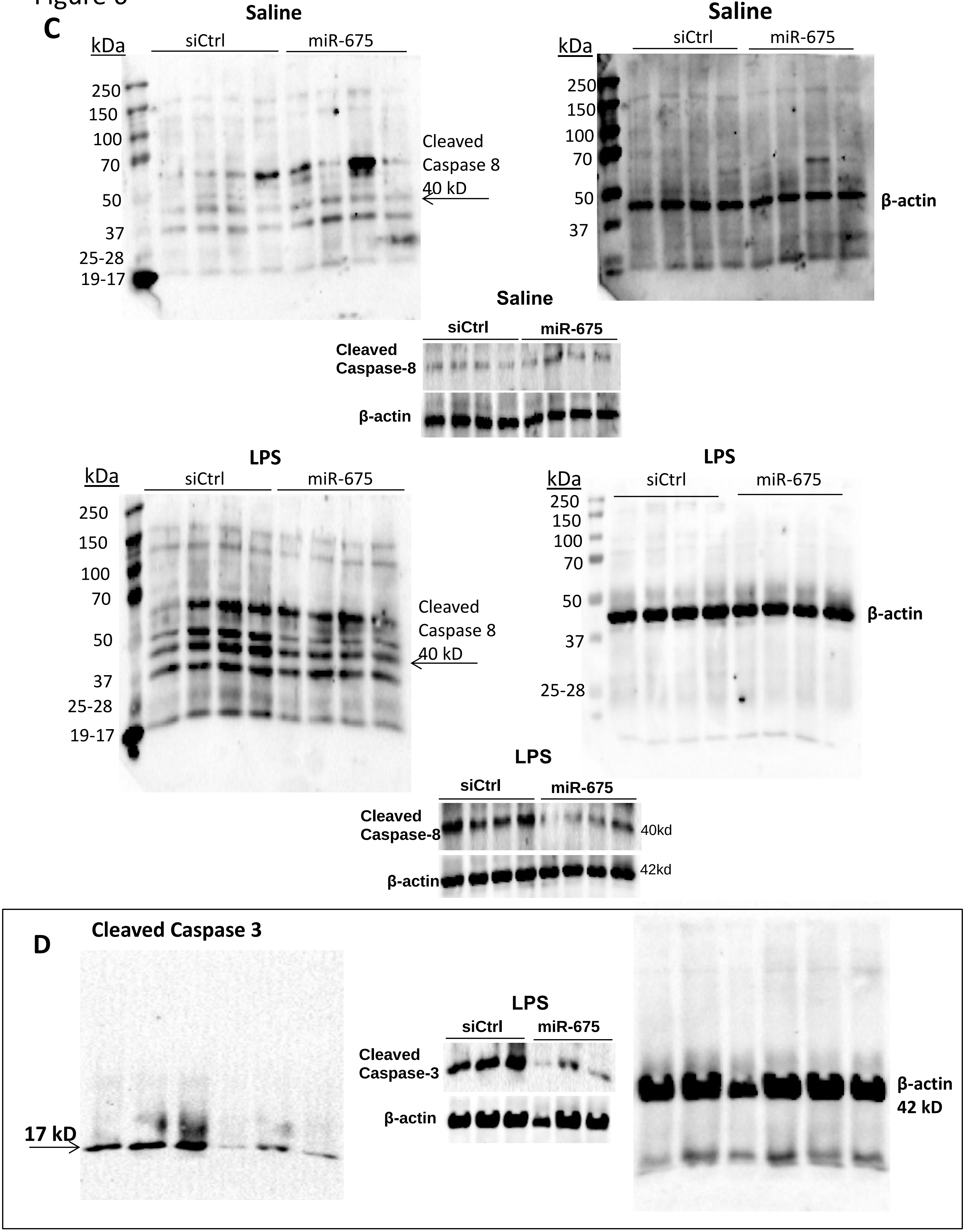
C
Saline
kDa
siCtrl
miR-675
250
150
100
70
50
37
25-28
19-17
Cleaved
Caspase 8
40 kD
Saline
kDa
siCtrl
miR-675
250
150
100
70
50
37
β-actin
Saline
siCtrl
miR-675
Cleaved
Caspase-8
β-actin
LPS
kDa
siCtrl
miR-675
250
150
100
70
50
37
25-28
19-17
Cleaved
Caspase 8
40 kD
LPS
kDa
siCtrl
miR-675
250
150
100
70
50
37
25-28
β-actin
LPS
siCtrl
miR-675
Cleaved
Caspase-8
40kd
β-actin
42kd
D
Cleaved Caspase 3
17 kD
LPS
siCtrl
miR-675
Cleaved
Caspase-3
β-actin
β-actin
42 kD

Figure 8

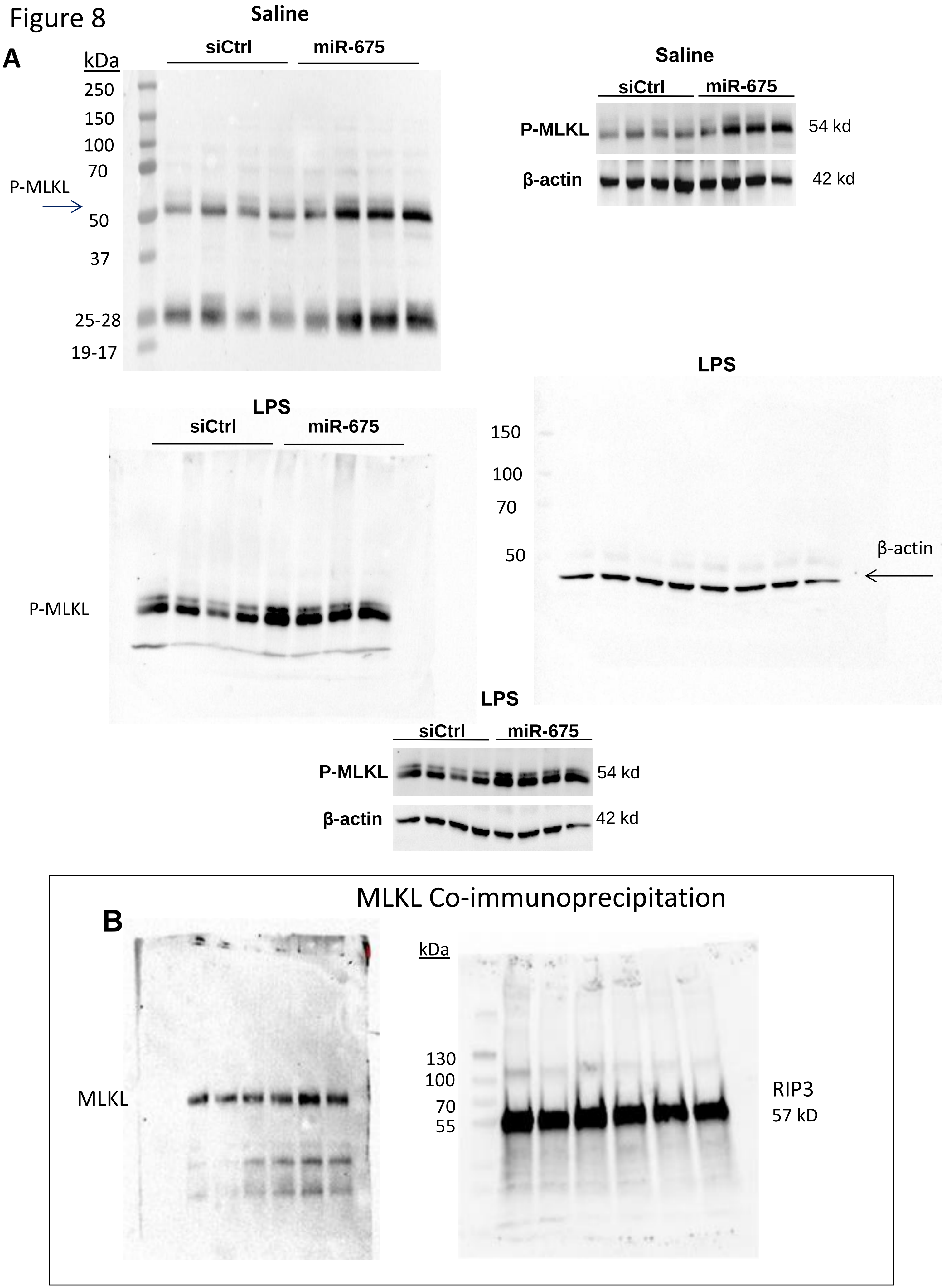
A
Saline
siCtrl
miR-675
kDa
250
150
100
70
P-MLKL
50
37
25-28
19-17
Saline
siCtrl
miR-675
P-MLKL
54 kd
β-actin
42 kd
LPS
siCtrl
miR-675
P-MLKL
LPS
150
100
70
50
β-actin
LPS
siCtrl
miR-675
P-MLKL
54 kd
β-actin
42 kd
MLKL Co-immunoprecipitation
B
MLKL
kDa
130
100
70
55
RIP3
57 kD
